# Supplementary material for: Experimental evolution reveals a general role for the methyltransferase Hmt1 in noise buffering
Source: PLoS Biol. 2019 Oct 15;17(10):e3000433. doi: 10.1371/journal.pbio.3000433 (PMC6814240; doi:10.1371/journal.pbio.3000433)

The signals are captured by a camera of UVP system.
The upper panel is the raw data of S2B Fig. In the panel, the right blot is hybridized with anti-GFP antibody to detect Tdh2-GFP and the left is with anti-G6pdh as loading control.
The bottom panel merges a photo for protein markers (captured at bright field; the most left lane in the left panel and the most right lane in the right panel) with an inversed shortlier-exposed photo of the upper panel.

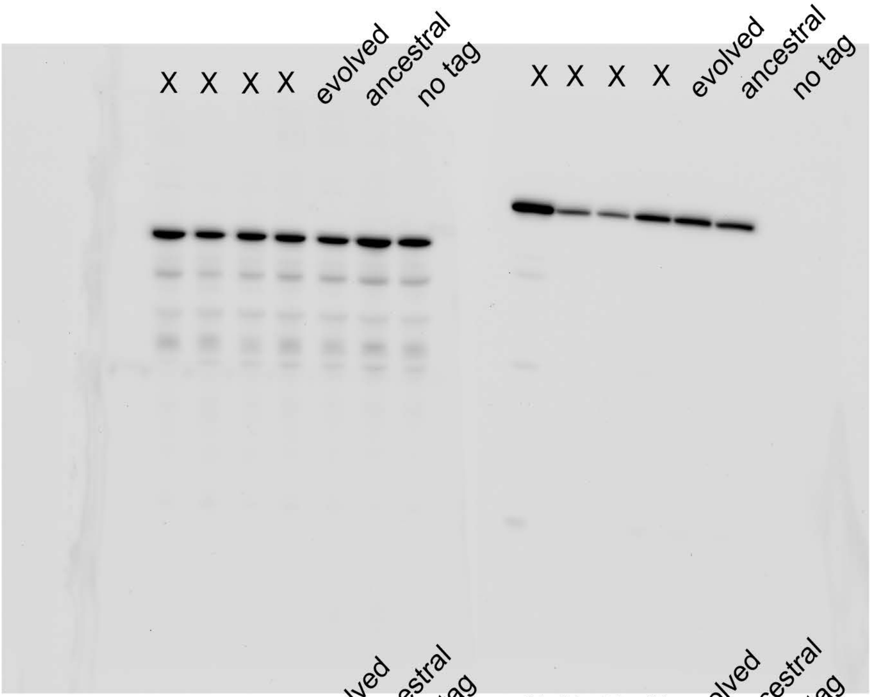

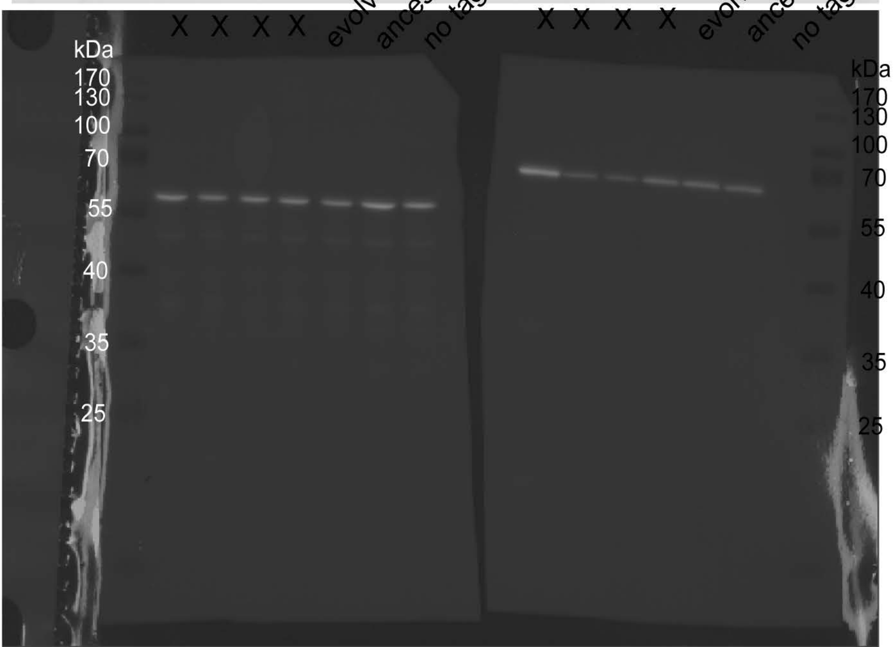

The signals are captured by a camera of UVP system.
The upper panel is the raw data for the left panel of S4C Fig.
The bottom panel merges a photo for protein markers
(captured at bright field; the most left lane) with the upper panel.

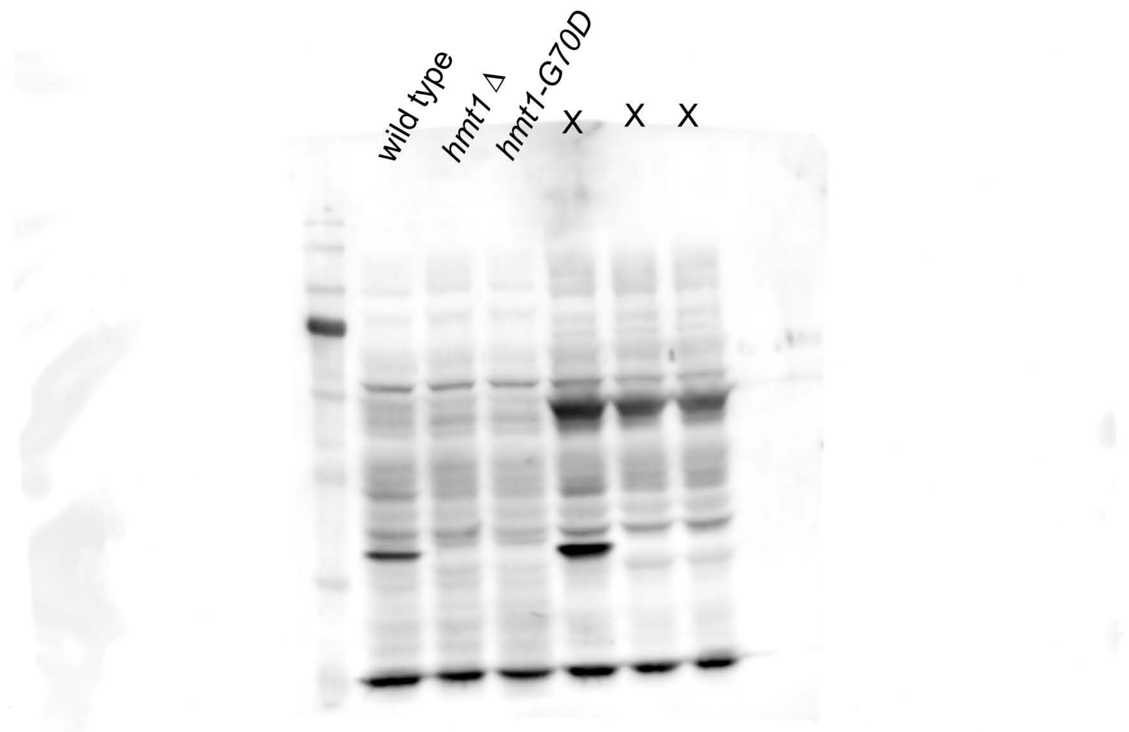

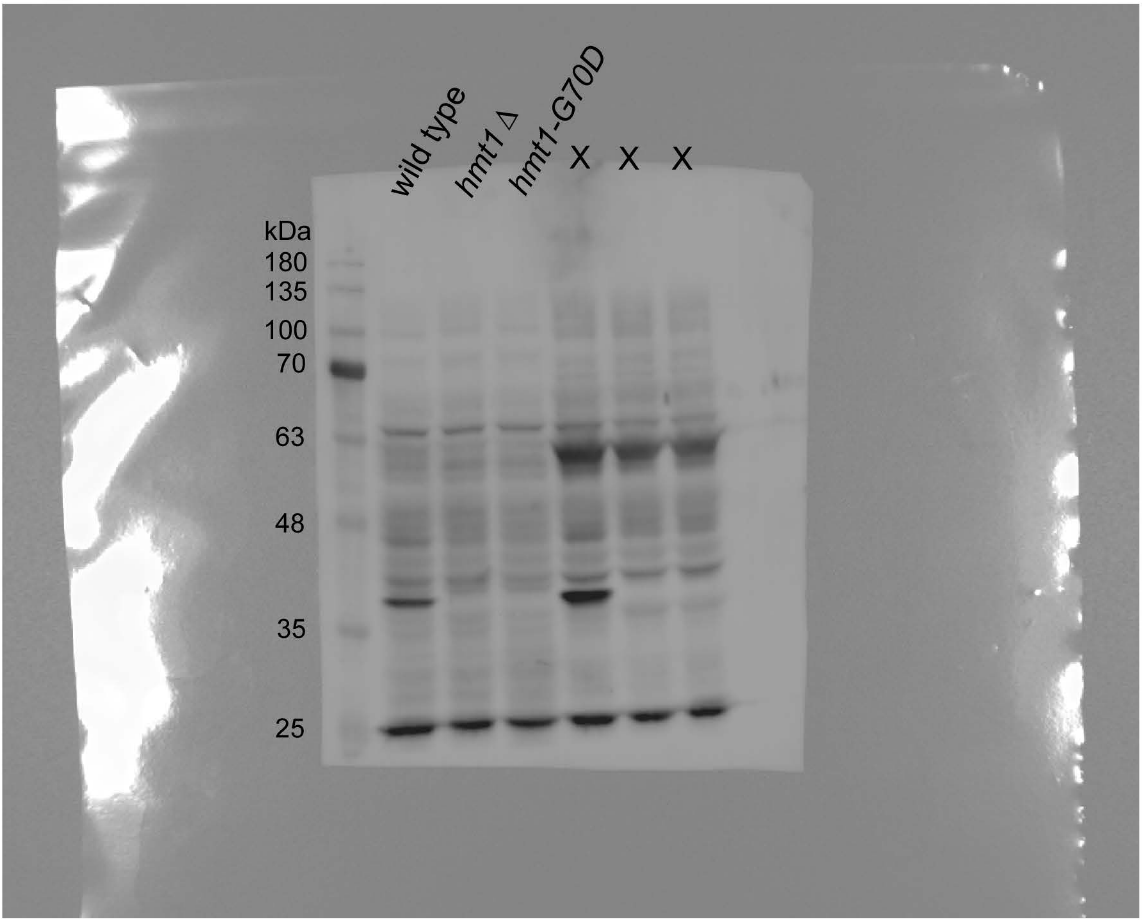

The signals are caputred by a X-ray film, and then the protein marker is labeled on the film (the most right lane). The photo is the raw data of the right panel of S4C Fig.

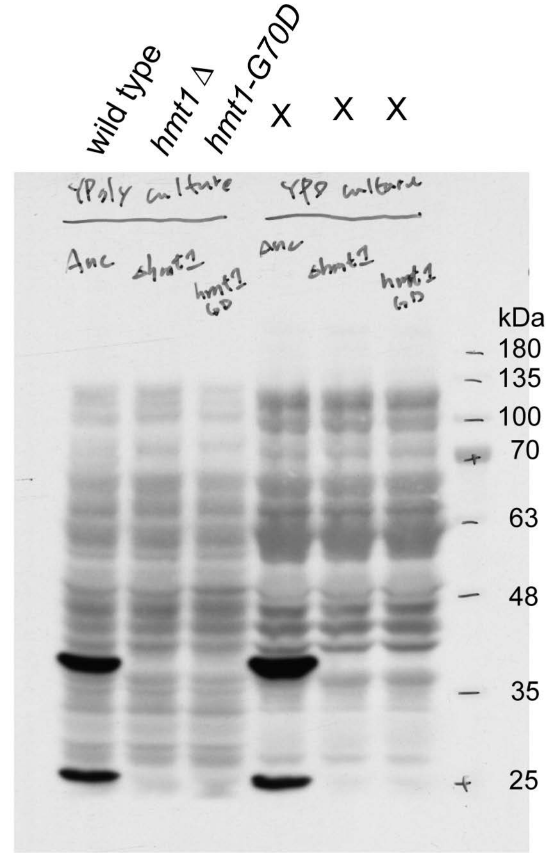

Supplement: S1 Raw Images — (PDF) [file pbio.3000433.s010.pdf]
