# Supplementary material for: Experimental evolution reveals a general role for the methyltransferase Hmt1 in noise buffering
Source: PLoS Biol. 2019 Oct 15;17(10):e3000433. doi: 10.1371/journal.pbio.3000433 (PMC6814240; doi:10.1371/journal.pbio.3000433)
Supplement: Attachment — Submitted filename: reply_to_reviewers.docx [file pbio.3000433.s011.docx]

1. Response to Editor (Editor’s comments in bold, responses in red):

**1.1 Based on the reviews, we will likely accept this manuscript for publication, providing that you will modify the manuscript according to the review recommendations. We have discussed Reviewer 1's requests, relating to the exact biochemical mechanisms and the evolutionary implications, with the Academic Editor. While we consider them very helpful, we will not require additional data to address these points as we acknowledge they go beyond the scope of the current study and do not affect the main conclusions. Therefore please address these points by toning down the relevant arguments, especially the putative links between HMT1-TDH2-bet hedging.**

We are grateful for the editor’s understanding and have toned down the relevant arguments in the revised manuscript (page 2, line 12; page 5, line 3; page 20, line 6).

**1.2 Note that we do not require all raw data. Rather, we ask that all individual quantitative observations that underlie the data summarized in the figures and results of your paper be made available in one of the following forms: 1) Supplementary files (e.g., excel). Please ensure that all data files are uploaded as 'Supporting Information' and are invariably referred to (in the manuscript, figure legends, and the Description field when uploading your files) using the following format verbatim: S1 Data, S2 Data, etc. Multiple panels of a single or even several figures can be included as multiple sheets in one excel file that is saved using exactly the following convention: S1_Data.xlsx (using an underscore). 2) Deposition in a publicly available repository. Please also provide the accession code or a reviewer link so that we may view your data before publication. Thank you for depositing you whole-genome sequence data at NCBI BioProject (PRJNA552720). Please also ensure that you provide the individual numerical values that underlie the summary data displayed in all the following figure panels as they are essential for readers to assess your analysis and to reproduce it: Figs. 1B, 2A-C, 3AB, 4A-C, 5A-E, 6A-D, S1, S2A, S3, S4B, S5**

Now we have provides all the raw data in an excel file “S1_Data”.

**1.3 For manuscripts submitted on or after 1st July 2019, we require the original, uncropped and minimally adjusted images supporting all blot and gel results reported in an article's figures or Supporting Information files.**

The original raw images of S2B and S4C Figs. with annotations are now provided in a pdf file named “S1_raw_images”.

2. Response to Reviewer 1 (Reviewer’s comments in bold, responses in red):

**The manuscript from You et al. describes a laboratory evolution experiment where population of yeast cells expressing a fluorescently-tagged enzyme were subjected to selection alternating for its high and low expression, respectively. The authors established evolved lines where noise in expression of the reporter protein ('protein noise') is increased as compared to the ancestor strain. They identify a mutation in HMT1 that largely contributes to this effect. They show that this mutation impairs the methyl-transferase activity of the enzyme, that it increases expression noise of various reporters (from related as well as unrelated pathways), and that ablation of HMT1 activity can increase expression noise not only in S. cerevisiae but also in S. pombe. All these demonstrations are nicely presented, with rigorous controls and convincing experimental results and I wish to congratulate the authors for the amount and quality of the work and for these findings.

The authors also address two other points in the manuscript and I see important issues in each one of them.

2.1 The first point is the mechanism of action of HMT1 on noise. Given the large spectrum of protein substrates that HMT1 modifies, this is a difficult question. The authors tested the effect of mutating four known targets (null or hypomorphic alleles), and they found an increase in noise for two mutants (rps2 and snf2), a decrease for one mutant (npl3) and no effect for the fourth mutant (sbp1). They tested the activation of CHA1, for which Swi/Snf is needed, in a hmt1-G70D mutant and found that is deficient. They also report that the noise increase of Tdh2::GFP and Gly1::GFP is accompanied by an increase of nucleosome occupancy at their promoters in the hmt1-G70D mutant. They conclude that Rps2 and SWI/SNF complex are relevant targets of HMT1 in controlling noise.
The data is of course consistent with this, but it is not a demonstration. For this claim (sentence 'is primarily achieved via two Hmt1 methylation targets' in abstract) to be demonstrated, the authors should perform double-mutant analysis: if the hmt1-G70D does not further increase noise in a snf mutant, and does not further reduce CHA1 activation in a snf mutant, then yes, SNF is needed for the effect of HMT1. If it does, then there are other targets that mediate the effect of HMT1.
Also, in all these analyses, the mean expression level of the reporter must be presented. Was it affected in some of the mutants?**

We agree that Snf2 and Rps2 may not be the only downstream targets of Hmt1 that affect noise buffering. We have rewritten the arguments in the revised manuscript (page 2, line 12; page 21, line 10).

Now we have provided both the mean and noise data in S1_Data. Some of the mutants did have slightly reduced mean values (as shown in the table below), but the expected effects on noise from the changes in the mean (based on the equation, “noise=variance/mean”, used in our study) are much less than the observed changes in the mutants that really increase noise (hmt1-G70D, rps2-DAMP, snf2, snf5 and snf6). We conclude that noise increases in these mutants are not simply due to the changes in the mean expression levels.

|  | ***hmt1 G70D*** | ***rps2*-DAMP** | ***snf2*Δ** | ***snf5*Δ** | ***snf6*Δ** | ***npl3*Δ** | ***sbp1*Δ** |
| --- | --- | --- | --- | --- | --- | --- | --- |
| **Fold change in the mean expression level** | 0.95 | 0.92 | 0.9 | 0.9 | 0.93 | 1 | 0.96 |
| **Expected fold changes in the noise from the fold changes in the mean based on the equation "noise = variance/mean"** | 1.05 | 1.09 | 1.11 | 1.11 | 1.08 | 1 | 1.04 |
| **Observed fold changes in the noise** | 1.4 | 1.29 | 1.72 | 1.56 | 2.46 | 0.87 | 1.03 |

**2.2** **The second point is whether HMT1 relays environmental sensing to expression noise, which would make it a fitness bet-hedging actor. The authors reach this conclusion on the basis of i) expression of HMT1 is reduced under stress (6A) ii) hmt1-G70D cells have a higher survival rate under stress (6C) iii) TDH2-GFP noise is higher under stress (6B) and iv) stationary TDH2-GFP-high cells better survive stress and re-germinate faster when re-fed (Fig 2). I agree that TDH2-GFP expression heterogeneity correlates with survival/fitness differences, but the data does not demonstrate that this happens via HMT1. If a change in HMT1 expression in response to stress modifies TDH2-GFP noise, then:
- forcing HMT1 expression to be constant (with a constitive promoter) despite stress should eliminate the effect of stress on TDH2-GFP noise and, possibly, decrease survival rate upon stress.
- TDH2-GFP noise should not increase (or not as much) upon stress in the hmt1-G70D mutant. The authors could simply repeat the experiment of Fig 6B and S5 on hmt1-G70D.**

**Without this, the action of HMT1 on noise is demonstrated but its implication on bet-hedging is not.**

(Jun-Yi, I may not answer it well, and the parts I manage to rewrite in the revised manuscript are also few.)

Since we did not perform the experiments suggested by the reviewer, we have toned down the role of Hmt1 in bet hedging in the revised manuscript (page 5, line 3; page 20, line 6).

**Other less-important recommendations:**

**2.3 The term "protein noise" is used throughout the article and must be rigorously defined in text.**

We have defined it in the introduction of the revised manuscript (page 3, line 4).

**2.4 Did the authors test if a drug inhibitor of HMT1, such as AMI-1, can increase noise?**

Thank the reviewer for this suggestion. We will test the drug in our future experiments.

**2.5 Fig2B: I see a linear decrease in the [10,17] mean GFP range and then a flat line at mean > 17. There could be a threshold at about ~17 where 'protection' is maximized.**

We appreciate the detailed observation of the reviewer. Indeed, the Tdh2-GFP intensity = 17 seems to be a threshold for maximized protection. However, the underlying mechanism is unclear so we did not further discuss this point.

**2.6 As written in discussion, the strong effect of snf6 deletion on noise was reported long ago (ref 11). This must also be cited in the results section (page 12 lines 9-11).**

We added this reference as requested.

**2.7 Fig7 tends to reduce the mechanistic model to Snf2 and Rps2 intermediate actors, while there are probably many others. This can mislead readers to focus their attention on these two HMT1 substrates only.**

We added “Protein X” in Fig. 7 to indicate that Snf2 and Rps2 may not be the only intermediate actors involved in this process.

**2.8 Fig S4C: meaning of arrowheads?**

We added the explanation in the figure legend.

**2.9 page 4, line 18: 'regularly face changing env' instead of 'constantly face changing env'?**

We think the reviewer means page 14, line18 (instead of page 4, line18). Since “regularly” may imply a fixed interval of time, we changed “constantly” to “frequently” instead (page 19, line 14)

**2.10 page7, line23: 'some populations', not 'same populations'**

We do mean “the same population”. The original sentence may be unclear, so we rewrote it into “…germination times among the individuals derived from the same population in some plants or fungi,…” (page 7, line 13).

**2.11 Fig6D: writing S. pombe or 'fission yeast' on the figure as a header would help.**

We added the header as the reviewer suggested.

3. Response to Reviewer 2 (Reviewer’s comments in bold, responses in red):

**In this paper You and Leu performed a yeast experimental evolution to find genes that regulate noise in gene expression. They started with a clever (though not very intuitive) strategy of repeated cycles of selection for cells that express tagged proteins highly and then lowly. After 35 such cycles they were able to increase level, without a trivial reduction in mean expression for two of the proteins. They speculated that they have achieved evolution for higher noise. They verified that the enhanced noise in the evolved strain was not specific to the gene that was used in evolution, indicating reassuringly that they have probably affected a general mechanism. They went on to sequence the genome and found a very high number of mutations (due to mutagen indeed) , but they followed up with a clever segregant analysis to scale down the number of candidates to a handful. They then found a hit, hmt1, and a single amino acid mutation in it, that appeared sufficient for noise enhancement (by putting the mutation in the wild-type, and by reverting it in the evolved strain). This methy-transferase appears to regulate noise through methylatin of diverse transcription and translation proteins, and itself it shows interesting response in the wild type to stress. This analysis nicely positions this gene as a central regulator in noise of yeast gene, as a mediator of bet-hedging. Strikingly this function was found to be conserved all the way to s pombe.

This is one of the most elegant papers I have seen in years in the study of noise of gene expression, and it provides one of the best results I have ever seen from experimental evolution! I congratulate the authors for super work. Everything they have done here was done masterfully, from the execution of the original evolution , to the very elegant segregant analysis, to their homing in on the regulators, and its characterization, and evolution.
Most importantly, the paper addresses and solves an important challenge - find regulators of noise, and their mechanism of action! few papers before have done something of this scale.

I could not find any potential "complaint" or advice. It never happened to me before as a referee.

I could have advised the authors to look genome wide at the G70D mutant to see if it affects noise of other genes, or if the effect is pathways specific. But I think they have a rounded story already now, and they (or others now) may leave that for the next one.
(I have blasted myself the protein againts other fungi and found that position 70 is very highly conserved, by that occasionally it's mutated (e.g. to Ala in Venturia inaequalis Colletotrichum fructicola), but never into an Asp as found here)
I thus enthusiastically recommend publication AS IS!**

We are grateful for the reviewer’s nice comments. Thanks.

4. Response to Reviewer 2 (Reviewer’s comments in bold, responses in red):

**This study investigates the role a methyltransferase in the buffering of “noise” in yeast. By performing microbial evolution experiments on S. cerevisiae to select for mutations that increase reporter protein noise, combined with bulk segregant analysis and CRISPR/Cas9-based reconstitution, the authors identified a molecular regulator of noise, namely methyltransferase Hmt1. Noise regulation was found primarily to be achieved via two Hmt1 methylation targets. Furthermore, Hmt1 was also found to buffer gene expression noise in S. pombe, a distant relative of S. cerevisiae, suggesting generality of the noise buffering mechanism. Overall, this is a highly relevant and timely study that has important implications for the fields of cellular/molecular biology and epigenetics, as well as for other related fields such as antifungal resistance research. Overall, the experiments are rigorous and support the main finding, the manuscript is well written, and the figures are of good quality. I support publication in PLOS Biology once the minor concerns below are addressed.**

**Minor Concerns**

**4.1 There are several works in the literature that I would like to see cited: 1) A review article on gene expression noise written by Kaern et al., Nat. Rev. Genet., 2005, which would provide a comprehensive introduction for readers unfamiliar with expression noise and its consequences. 2) When specifically discussing gene network topology and noise (e.g., on Page 5, Line: 11), it would be appropriate to cite Charlebois et al., Phys. Rev. E, 2014; it would also be relevant to cite a related study demonstrating that the properties of gene expression noise can evolve on the fitness landscape (Charlebois, Phys. Rev. E., 2015), for example, on Page: 5, Line 5. 4) Very recently, Farquhar et al., Nat. Commun., 2019 demonstrated that gene expression noise modulated by gene network topology can influence drug resistance and the selection/establishment of genetic mutations (relevant, e.g., on Page: 4, Lines: 8-9) - it would be interesting to discuss this work in the context of your findings.**

All the suggested references were added (Kaern et al., 2005 on page 3, line 21; Charlebois et al., 2014 and Charleboise et al., 2015, on page 4, lines 6 and 13; and as Farquhar et al., 2019, on page 21, line 23). We also added related discussion on page 21, line 21, as the reviewer suggested.

**4.2 Page 4: Lines 13-16. Perhaps this is just semantics, but I think it would be more accurate to say “complex regulatory networks that respond to randomly fluctuating environments…”, because if the environment fluctuates periodically then, for instance, oscillating gene regulatory network dynamics (which seem to have selected for in other instances, such as circadian rhythms) would confer a lower fitness cost than both sense/response networks and bet-hedging.**

We revised the manuscript as requested.

**4.3 Page 8: Lines 10-14. Be cautious about concluding that an observed phenomenon is nongenetic in nature from “relaxation” type experiments, as for example, it is possible that the mutants were instead outcompeted by “wild type” cells when the experimental conditions changed. The only way to ascertain for certain whether an adaptation is nongenetic or genetic is to perform sequencing (as you have done in the rest of the study).**

We agree with the reviewer and have toned down our conclusion (page 9, line 2; page 44, line 3).

**4.4 Avoid overstating your findings. For instance, on Page 14: Lines 13-15 I would say “may represent an important survival strategy…” instead of “probably represents an important survival strategy…” in the context of “diverse microorganisms”. Alternatively, I think it would be reasonable to say “probably represents an important survival strategy in yeasts”.**

As the reviewer suggested, we have changed the sentence to “may represent an important survival strategy”.

**4.5 Any scripts/codes used to analyze the experimental data and perform sequence simulation should be made is freely available on a site such as GitHub – this will ensure reproducibility of the results and maximize the utility of this work to the research community**

The script used for determining the threshold of allele frequency for causal mutations in bulk segregant analysis is uploaded to GitHub now. It can be found by searching “Threshold-for-bulk-segregant-analysis”, and the script is under a repository “imst0715”.
